# Effect of 2,3,7,8-Tetrachlorodibenzo-*p*-dioxin (TCDD) on Hormones of Energy Balance in a TCDD-Sensitive and a TCDD-Resistant Rat Strain

**DOI:** 10.3390/ijms150813938

**Published:** 2014-08-12

**Authors:** Jere Lindén, Sanna Lensu, Raimo Pohjanvirta

**Affiliations:** 1 Department of Veterinary Biosciences, Faculty of Veterinary Medicine, University of Helsinki, P.O. Box 66, FI-00014 Helsinki, Finland; 2 Department of Biology of Physical Activity, Faculty of Sport and Health Sciences, University of Jyväskylä, P.O. Box 35, FI-40014 Jyväskylä, Finland; E-Mail: sanna.lensu@jyu.fi; 3 Department of Environmental Health, National Institute for Health and Welfare (THL), P.O. Box 95, FI-70701 Kuopio, Finland; 4 Department of Food Hygiene and Environmental Health, Faculty of Veterinary Medicine, University of Helsinki, P.O. Box 66, FI-00014 Helsinki, Finland; E-Mail: raimo.pohjanvirta@helsinki.fi

**Keywords:** 2,3,7,8-tetrachlorodibenzo-*p*-dioxin, TCDD, wasting syndrome, energy balance, hormones, acute toxicity, strain differences, AHR

## Abstract

One of the hallmarks of the acute toxicity of 2,3,7,8-tetrachlorodibenzo-*p*-dioxin (TCDD) is a drastically reduced feed intake by an unknown mechanism. To further elucidate this wasting syndrome, we followed the effects of a single large dose (100 μg/kg) of TCDD on the serum levels of several energy balance-influencing hormones, clinical chemistry variables, and hepatic aryl hydrocarbon receptor (AHR) expression in two rat strains that differ widely in their TCDD sensitivities, for up to 10 days. TCDD affected most of the analytes in sensitive Long-Evans rats, while there were few alterations in the resistant Han/Wistar strain. However, analyses of feed-restricted unexposed Long-Evans rats indicated several of the perturbations to be secondary to energy deficiency. Notable increases in ghrelin and glucagon occurred in TCDD-treated Long-Evans rats alone, which links these hormones to the wasting syndrome. The newly found energy balance regulators, insulin-like growth factor 1 and fibroblast growth factor 21 (FGF-21), appeared to function in concert in body weight loss-induced metabolic state, and FGF-21 was putatively linked to increased lipolysis induced by TCDD. Finally, we demonstrate a reverse set of changes in the AHR protein and mRNA response to TCDD and feed restriction, suggesting that AHR might function also as a physiological regulator, possibly involved in the maintenance of energy balance.

## 1. Introduction

Dioxins are a group of pervasive and biomagnifying environmental contaminants that cause concern for their high acute toxicity and teratogenicity in laboratory animals [1,2,3], and putative developmental effects in humans at body burdens of low ng/kg levels [4,5]. These chemically heterogeneous compounds, generally represented in toxicity studies by the most potent member 2,3,7,8-tetrachlorodibenzo-*p*-dioxin (TCDD), share a common fundamental mechanism of action, conveying their effects through an intracellular aryl hydrocarbon receptor (AHR) [6]. Furthermore, structural variation in the AHR appears to be the major determinant of the astonishingly wide intra- and inter-species differences in susceptibility to dioxin toxicity [7]. This is exemplified in the vast TCDD-sensitivity difference between two rat strains: The LD_50_ value for TCDD of extremely resistant Han/Wistar (*Kuopio*; H/W) rats, carrying an AHR mutation, is about 1000 times higher than the TCDD LD_50_ value of Long-Evans (*Turku/AB*; L-E) rats and a similar disparity of response is also demonstrable in many, but not all, toxicity end-points.

The principal features of acute TCDD toxicity, besides inter- and intra-species differences in susceptibility, are delayed lethality and drastic and persistent changes in body weight and feed intake [8,9]. Even high acute dioxin doses do not kill animals immediately, but death occurs in one to several weeks, and in many species (e.g., rats, mice, hamsters and guinea pigs), after substantially reduced feed intake and prominent weight loss [10,11,12,13], called the wasting syndrome. The mechanism of this derailment of body weight regulation has largely remained elusive in spite of over three decades of research effort.

The endocrine system acts as a major regulator and mediator of energy balance in the body, but its role in the wasting syndrome is poorly known. In the present study, we aimed to elucidate that aspect of the wasting syndrome employing the TCDD-sensitivity difference of the resistant H/W and sensitive L-E rats. We used a high dose of TCDD (100 µg/kg) which would ultimately (in 3–6 weeks) have been lethal to all L-E rats but sublethal to all H/W rats [1]. Although this represents a far higher exposure to TCDD compared with background exposure of the general public, it is scientifically justified, not only because it furthers our understanding of the wasting syndrome but also because it provides important information of the effects that may emerge when people are exposed to exceptionally high doses of dioxins in accidents [14,15] or poisonings [16]. We determined the effects of a single dose of TCDD and comparable feed restriction (mimicking the body weight loss in the wasting syndrome) for up to 10 days on the serum levels of selected hormones and products/substrates of intermediary metabolism that are known to participate in, or affect, peripheral and central regulation of energy balance. The measured metabolism-related hormones were insulin, leptin, ghrelin, glucagon, adiponectin, fibroblast growth factor 21 (FGF-21), insulin-like growth factor 1 (IGF-1), and corticosterone, and the metabolites/substrates were glucose, free fatty acids (FAA), triglycerides, cholesterol, and 3-hydroxybutytyrate (β-hydroxybutyrate, BHB). In order to compare the consequences of TCDD-administration and feed restriction for liver, we assessed the extent of liver damage by measuring serum alanine aminotransferase (ALAT) and aspartate aminotransferase (ASAT), and explored the changes in AHR expression at mRNA and protein levels.

The principal signals mediating sensitivity to meal-associated satiety signals are adipocyte-derived leptin and pancreatic insulin, which convey information about body adiposity to the central nervous system (CNS) [17,18,19], while insulin also has a second, partly overlapping and equally important role in constant regulation of plasma glucose by stimulating glucose uptake and anabolic processes in several tissues, and by inhibiting glycogenolysis and gluconeogenesis in the liver [20]. Depletion of leptin or insulin, or their receptors, generally results in increased food intake and obesity mediated by the brain, largely arising from lowered sensitivity to meal-generated satiety signals [19,21,22]; type 1 diabetes represents a pathological state of insulin depletion leading to weight loss due to severely impaired glucose utilization and reduced ability of adipocytes to store fat. Administration of exogenous leptin or insulin (at low doses not inducing hypoglycaemia) brings about (at least short-term) reductions in food intake and body weight [23,24,25,26,27].

Ghrelin is the sole generally accepted, primarily peripheral hormonal signal directly inducing food intake and seems to act both as an “acute” hunger signal and as a longer-term augmenting factor of feeding [28,29,30,31]. It is produced both peripherally (mainly in the stomach) and centrally, and its primary functions are the secretion of growth hormone from the anterior pituitary cells and the central regulation of energy homeostasis through the modulation of appetite and food intake. Ghrelin appears to increase food intake by direct CNS receptor stimulation and/or through the vagus nerve, with the possible involvement of hypothalamic *de novo* synthesis of this hormone [28,29,30,32,33,34,30,32].

Besides the above two principal (most studied) adiposity signals [35], pancreatic glucagon, produced in α-cells of the pancreas, and adiponectin, produced by adipose tissue, have quite recently attracted attention as regulators of metabolism and eating, while the adrenal glucocorticoids have long been known to act peripherally as insulin antagonists. Glucagon has a major role maintaining the blood glucose within tight limits and responding to hypoglycemia by increasing hepatic gluconeogenesis [36,37]; it has also received renewed interest in the pathogenesis of diabetes [38]. Despite the peripheral insulin antagonism, glucagon, however, reduces eating akin to leptin and insulin, acting either via the liver—vagus—*nucleus tractus solitarius* pathway or by directly binding to CNS receptors, and shows an additive anorexigenic effect with glucagon like peptide 1 [19,27,39]. Adiponectin acts as a pivotal peripheral signal to increase insulin sensitivity [40], while it has been reported to either acutely increase food intake and activate AMP-activated protein kinase in the mouse hypothalamus [41], or slightly reduce eating after chronic central administration to spontaneously hypertensive rats [42]. Glucocorticoids promote liver glucose output by activating gluconeogenesis, especially in response to fasting and stress, while at the same time triggering insulin resistance, inhibiting glycogen synthesis and promoting protein catabolism in the muscle [43,44]. They also increase food intake by stimulating hypothalamic circuits, by acting as a permissive factor for the orexigenic neuropeptide-Y [45], and by inhibiting secretion of the anorexigenic corticotropin-releasing hormone [35].

Liver is the primary source of two polypeptides, FGF-21 and IGF-1, that have lately been discovered to act as hormones regulating peripheral metabolism. FGF-21 is produced also in other tissues involved in glucose and lipid metabolism such as adipose tissue, pancreas and skeletal muscle [46,47,48], and it acts in a bidirectional manner through incompletely understood mechanisms (reviewed in [49,50]). In fasting, FGF-21 functions as a hormonal starvation signal (regulated through peroxisome proliferator-activated receptor α [PPARα] in the liver) stimulating gluconeogenesis, fatty acid oxidation, ketogenesis, and torpor [51,52,53], while it is also strongly induced by feeding and obesity. In energy excess, FGF-21 enhances PPARγ activity and adipogenesis in white adipose tissue as an autocrine/paracrine factor, concurrently systemically reversing hepatic steatosis, increasing energy expenditure, and improving insulin sensitivity [54,55,56]. IGF-1 is mainly produced in hepatocytes under growth hormone (GH) stimulation [57,58]. Its principal effect on intermediary metabolism is increasing insulin sensitivity, apparently both through direct binding to type 1 IGF receptor and suppression of GH (a potent insulin antagonist) secretion through a negative feedback loop [58,59]. Intriguingly, in starvation GH concentrations are increased, but its anabolic actions and induction of IGF-1 are lost through GH resistance, likely mediated, at least in part, by FGF-21 [49,60,61].

Peripheral hormonal signals directly inducing food intake are conspicuously few, but several neurons in the CNS and periphery are capable of sensing the levels of glucose, free fatty acids (FFAs) and amino acids [22,62,63]. Direct sensing of foremost glucose maintains the short-term energy stores at physiological levels and a decrease below the euglygemic level triggers an immediate peripheral response starting with the secretion of glucagon, epinephrine, and norepinephrine [63]. In advanced, non-physiological, acute energy depletion states this is followed by initiation of feeding [36,64]. In parallel, sensing of lipids in the intestine as well as of FFAs and amino acids in the brain lowers hepatic glucose production and appears to reduce food intake [62,65,66]. Ketone bodies (acetone, acetoacetic acid, and BHB) are produced in the liver from fatty acids for energy transportation after muscle and liver stores of glycogen are depleted. They are currently under active study for their signaling functions and possible effects on longevity [67]. Ketogenic, low-carbohydrate diets reduce weight in humans, but the exact mechanisms of the weight loss are uncertain [68].

## 2. Results and Discussion

Our aim was to investigate the effects of a single dose of TCDD on serum levels of hormones and products/substrates of intermediary metabolism influencing peripheral and CNS regulation of energy balance. We used a TCDD dose (100 μg/kg) that generates the typical wasting syndrome in L-E rats but induces only a slight and reversible weight reduction in H/W rats, and tried to isolate the TCDD-effects from secondary effects of energy depletion by employing a feed-restricted L-E control group. The TCDD-treated H/W and L-E rats were sampled at 1, 4 and 10 days and feed-restricted L-E rats at 4 and 10 days. In addition to the energy metabolism-related measurements, effects on the liver by TCDD and feed restriction were assessed through quantification of serum concentrations of the hepatic indicator enzymes ALAT and ASAT. Moreover, as AHR is indispensable to all major toxicities of TCDD [6], mRNA and protein expression levels of AHR in the liver were determined. The results are depicted below, grouping related parameters together, followed by a collective discussion.

### 2.1. Body Weight Change

**Figure 1:**
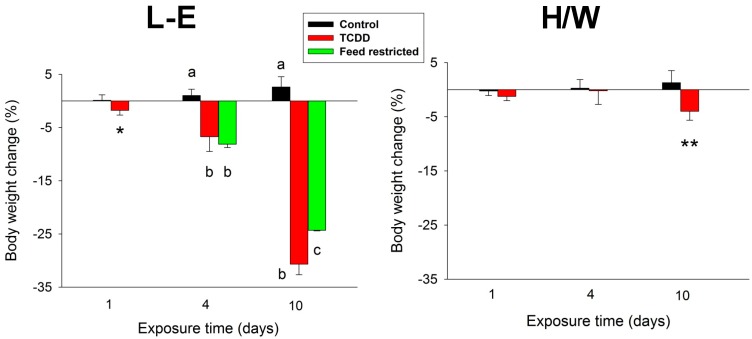
The effect of TCDD or feed restriction on body weight (mean ± SD) in L-E (**left** panel) and H/W rats (**right** panel) on days 1, 4 and 10 (*n* = 5, except in the H/W strain on day 1 where *n* = 4). Columns with different letters at a single time-point differ significantly from one another (*p* < 0.05). The asterisks denote a statistically significant difference (*t*-test) from the corresponding control (L-E rats on day 1 and the H/W strain at all time-points) as follows: * = *p* < 0.05; ** = *p* < 0.01; *** = *p* < 0.001.

As expected, in L-E rats TCDD caused a striking, progressive body weight loss, which amounted to about 30% of initial body weight by day 10 (Figure 1). In feed-restricted control L-E rats, body weight decreased to the same extent at 4 days but somewhat less (approximately 25%) at 10 days. H/W rats, in contrast, were resistant to the TCDD-induced wasting as evidenced by their marginal (<5%) reduction of body weight at the end of the experiment (Figure 1).

### 2.2. Leptin, Insulin, Glucagon, and Adiponectin Serum Concentrations

**Figure 2:**
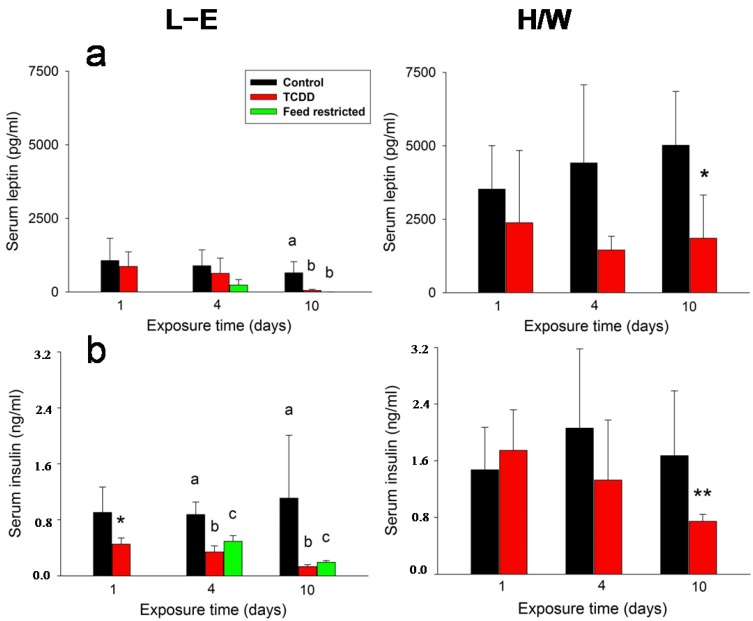
The effect of TCDD or feed restriction on (**a**) serum leptin and (**b**) serum insulin concentrations in L-E (**left** panel) and H/W rats (**right** panel) as a function of time (mean ± SD). The conditions are as in Figure 1.

The serum concentrations of both leptin (Figure 2a) and insulin (Figure 2b) generally followed the decrease of body weight with both hormones showing reduced levels during the observation period. However, in L-E rats the change in circulating insulin concentrations was statistically significant at all time-points whereas for leptin, significance was not reached until day 10. Feed-restricted controls responded in a similar way, but, in the case of insulin, the reduction remained milder. There was a notable strain difference in leptin and insulin serum concentrations, since these were about three or two times higher, respectively, in H/W *vs.* L-E rats. In the H/W strain, TCDD caused moderately decreasing trends in leptin and insulin concentrations, which attained statistical significance at day 10.

**Figure 3:**
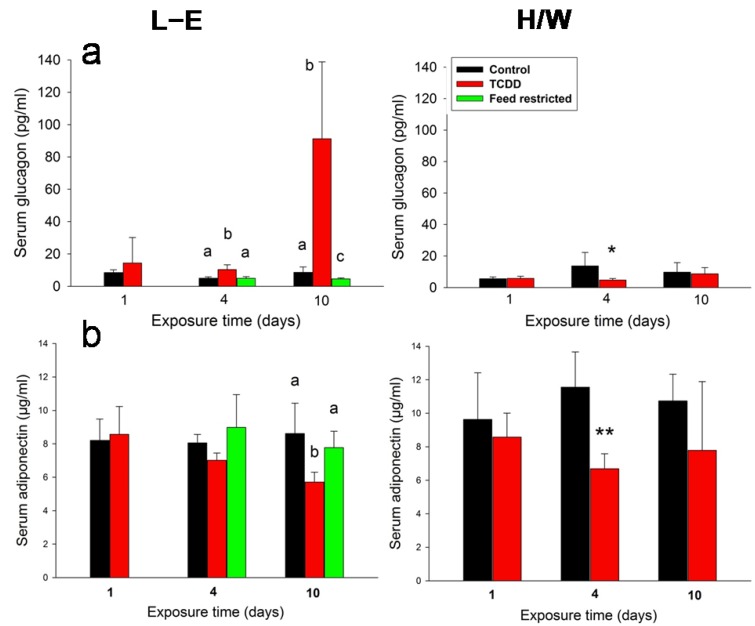
The effect of TCDD or feed restriction on (**a**) serum glucagon and (**b**) adiponectin concentrations in L-E (**left** panel) and H/W rats (**right** panel) as a function of time (mean ± SD). The conditions are as in Figure 1.

In L-E rats, serum glucagon concentration (Figure 3a) exhibited a slight, but statistically significant increase at 4 days and a substantial (approximately 10-fold) increase at 10 days, the latter concurrently with a meager reduction in the feed-restricted group. No such increase was observable in H/W rats; in fact, a small drop was recorded on day 4. Adiponectin levels (Figure 3b) were diminished by TCDD at 4 or 10 days in H/W and L-E rats, respectively. Feed restriction did not affect this hormone’s concentrations.

### 2.3. IGF-1, Ghrelin, Corticosterone and FGF-21 Serum Concentrations

**Figure 4:**
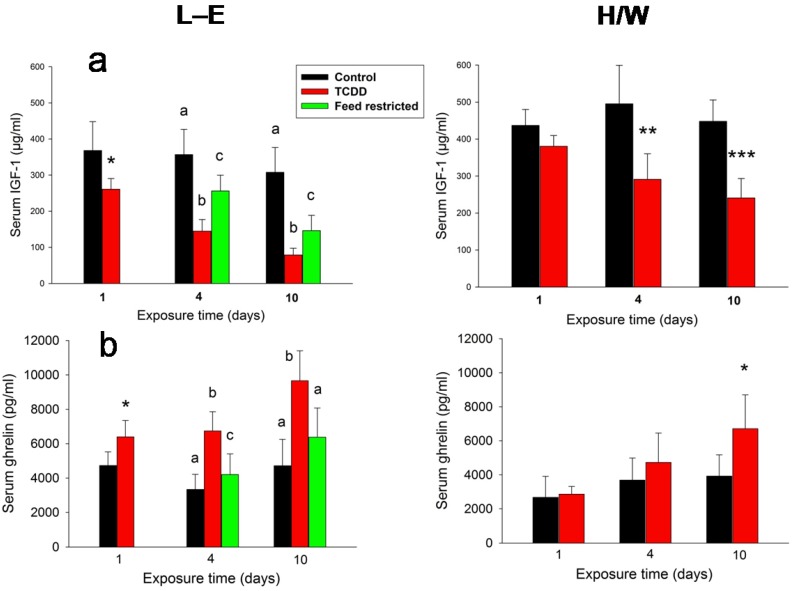
The effect of TCDD or feed restriction on (**a**) serum IGF-1 and (**b**) ghrelin concentrations in L-E (**left** panel) and H/W rats (**right** panel) as a function of time (mean ± SD). The conditions are as in Figure 1.

TCDD induced a stark, rapid and progressive reduction of serum IGF-1 concentration (Figure 4a) in both L-E and H/W rats, although the effect was less pronounced in the latter. Feed-restricted L-E rats responded in a qualitatively similar manner, but to a significantly lower degree than their TCDD-treated counterparts. Ghrelin serum concentrations (Figure 4b) displayed changes that were essentially a mirror-image of those of IGF-1: a rapid, progressive increase in TCDD-treated rats with the response being stronger in L-E than H/W rats, and a more lenient elevation in feed-restricted controls.

**Figure 5:**
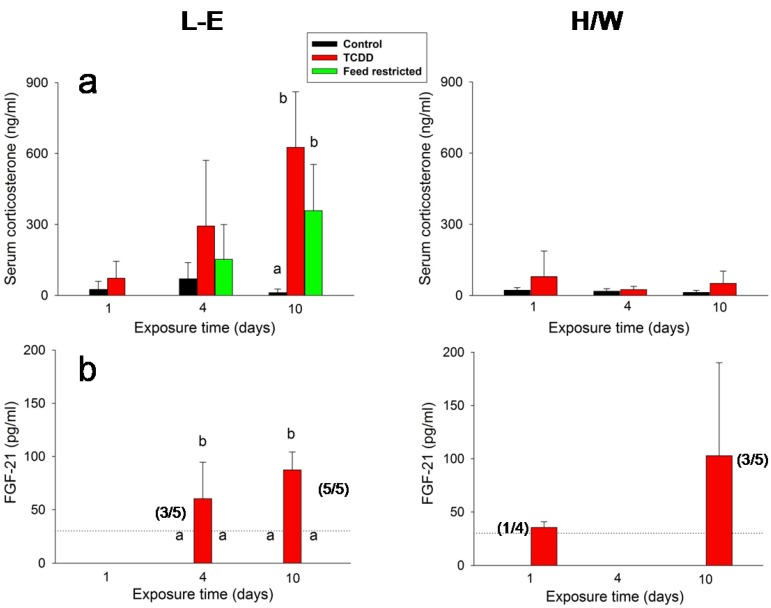
The effect of TCDD or feed restriction on (**a**) serum corticosterone and (**b**) FGF-21 concentrations in L-E (**left** panel) and H/W rats (**right** panel) as a function of time (mean ± SD). In (**b**) the FGF-21 assay detection limit, corresponding to a serum concentration of 30 pg/mL, is depicted with a horizontal dotted line. The number of animals exceeding the limit per total group size (4–5) is given in parenthesis. For those groups not shown in the graph, all values fell below the detection limit. The height of the bar depicts the mean of all animals in the group. The means and SDs are calculated by setting the below threshold values to 30 pg/mL. Otherwise, the conditions are as in Figure 1.

In L-E rats, serum corticosterone concentrations (Figure 5a) exhibited an upward trend after both TCDD administration and feed restriction on day 4, and a substantial (30–50-fold) increase after both treatments on day 10. The increments brought about by TCDD were at both time-points larger than those generated by feed restriction; however, the measurements displayed large variations and there was no significant difference between them. Contrary to L-E rats, TCDD did not induce changes to serum corticosterone concentration in the H/W strain.

Among the most divergent responses elicited by TCDD *vs.* feed restriction were those in FGF-21 serum concentrations (Figure 5b). TCDD administration appeared to induce a progressive increase in FGF-21 secretion starting on day 4 in L-E rats, and a similar but less pronounced response in the H/W strain. There was a statistically significant increase in the FGF-21 serum concentration in three of the five TCDD-treated L-E rats at 4 days (*n* = 3, mean = 80 pg/mL) and in all five TCDD-treated L-E rats at 10 days (*n* = 5, mean = 87 pg/mL). In addition to these animals, values exceeding the assay detection limit were recorded in one out of four TCDD treated H/W rats on day 1 (40 pg/mL) and in three of the five TCDD-treated H/W rats at 10 days (*n* = 3, mean = 151 pg/mL). In all *ad libitum* control L-E and H/W rats as well as in feed-restricted L-E animals, serum FGF-21 concentrations fell below the assay limit.

### 2.4. Clinical Chemistry and AHR

**Figure 6:**
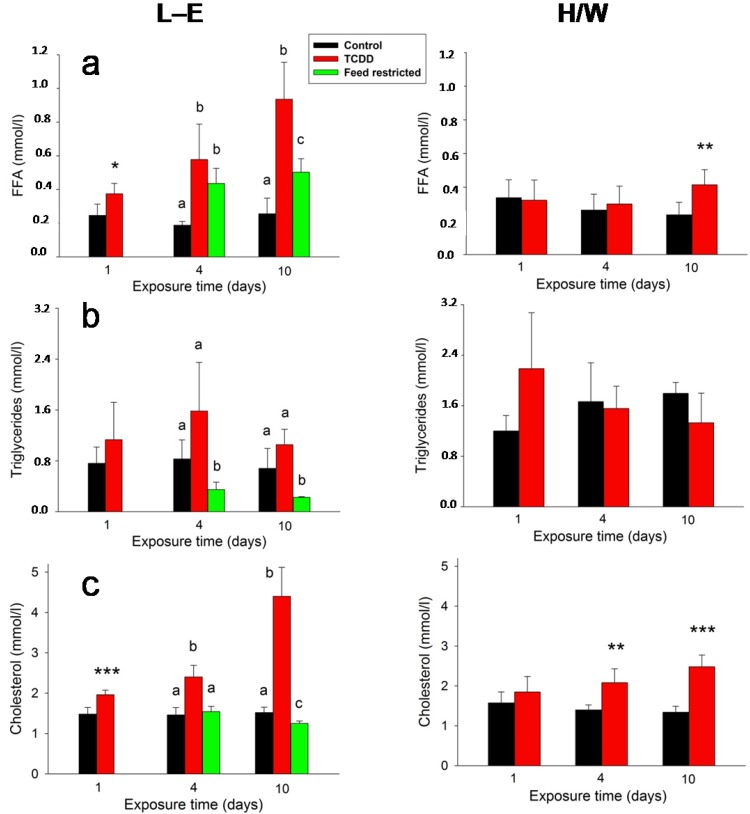
The effect of TCDD or feed restriction on (**a**) serum FFA; (**b**) triglyceride and (**c**) cholesterol concentrations in L-E (**left** panel) and H/W rats (**right** panel) as a function of time (mean ± SD). The conditions are as in Figure 1.

Cholesterol (Figure 6a) and FFA (Figure 6b) serum concentrations exhibited a similar response to TCDD in L-E rats: there was a statistically significant increase already on day 1 and the difference to the control group progressively expanded on days 4 and 10. In comparison, while feed reduction had a statistically significant increasing effect on FFA on days 4 and 10 (which was significantly smaller than the elevation induced by TCDD on day 10), it induced a slight reduction of cholesterol on day 10. In H/W rats, TCDD brought about a small, but statistically significant increase in FFA on day 10 and progressive, albeit more subdued, increase in cholesterol. Contrary to the previous lipoid parameters, TCDD did not alter triglyceride (Figure 6c) serum concentrations either in L-E or in H/W rats, but feed restriction induced a marked reduction both on day 4 and day 10.

**Figure 7:**
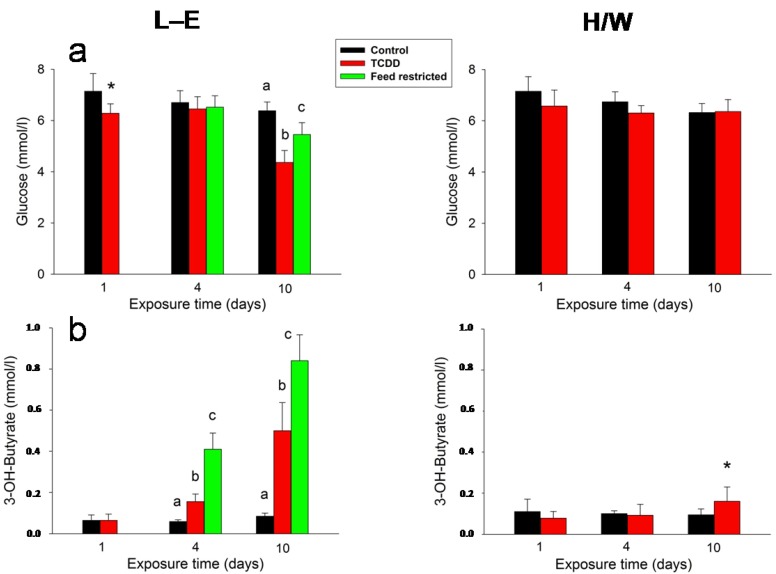
The effect of TCDD or feed restriction on (**a**) serum glucose and (**b**) BHB concentrations in L-E (**left** panel) and H/W rats (**right** panel) as a function of time (mean ± SD). The conditions are as in Figure 1.

Glucose (Figure 7a) and BHB (Figure 7b) were not considerably affected by TCDD in H/W rats (although there was a marginal, statistically significant increase in BHB at 10 days). In contrast, in L-E rats the BHB serum concentrations demonstrated an advancing ketonemia on days 4 and 10 in both TCDD-treated and feed-restricted groups, and there was also a statistically significant reduction in serum glucose in both groups on day 10. However, serum glucose concentration was significantly lower at 10 days in the TCDD-treated than in the feed-restricted group, while, counter-intuitively, the BHB increase was significantly more pronounced after feed restriction than after TCDD administration at both 4 and 10 days.

**Figure 8:**
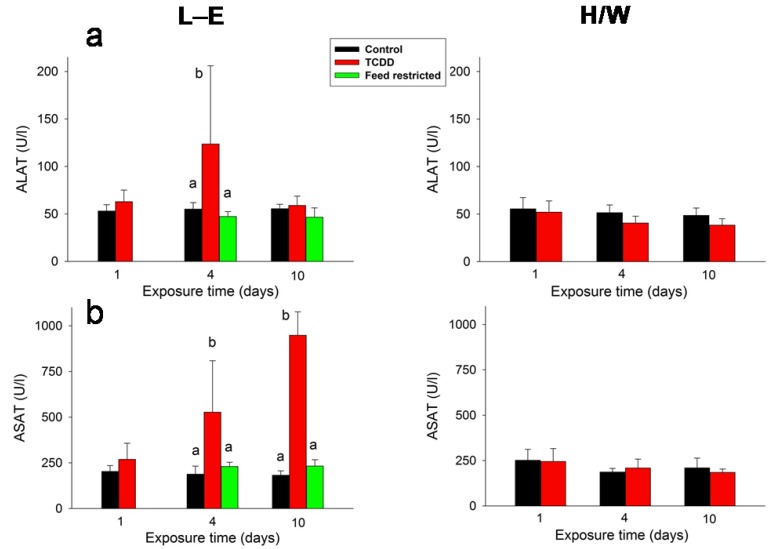
The effect of TCDD or feed restriction on (**a**) serum ALAT and (**b**) ASAT concentrations in L-E (**left** panel) and H/W rats (**right** panel) as a function of time (mean ± SD). The conditions are as in Figure 1.

ALAT (Figure 8a) was transiently doubled on day 4 in TCDD-dosed L-E rats, whereas ASAT (Figure 8b) exhibited a progressive increase at 4 (two-fold) and at 10 (four-fold) days after TCDD administration. Feed restriction did not have any effect on the transaminases, nor were they affected by TCDD in H/W rats.

**Figure 9:**
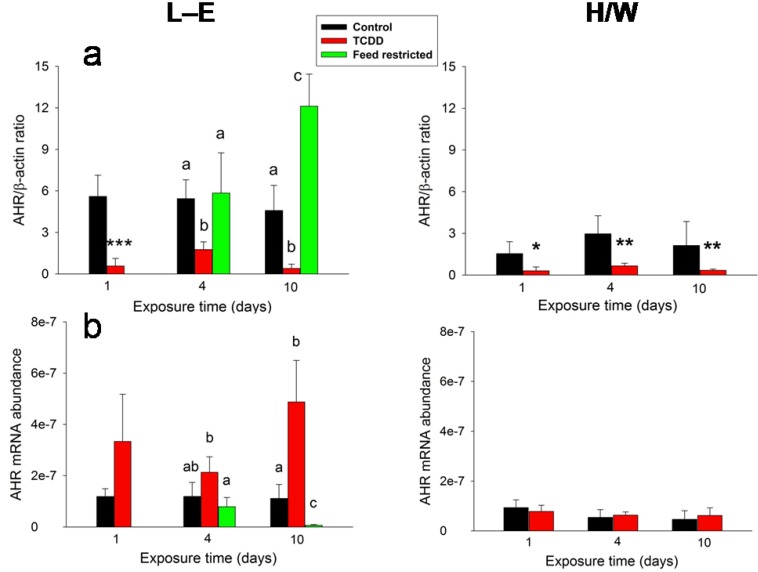
The effect of TCDD or feed restriction on liver AHR (**a**) protein and (**b**) mRNA levels in L-E (**left** panel) and H/W rats (**right** panel) as a function of time (mean ± SD). The conditions are as in Figure 1.

In L-E rats, TCDD administration and feed restriction resulted in antithetical AHR protein (Figure 9a) and mRNA (Figure 9b) abundance changes in the liver, which again were in opposite directions after the two treatments. TCDD induced a marked reduction of AHR protein at all time-points in both rat strains, whereas feed restriction brought about essentially no change in the protein level on day 4, but an over two-fold increase on day 10. AHR mRNA abundance was affected in a totally disparate manner. In L-E rats, there was an upward tendency at 1 and 4 days and an approximately four-fold increase at 10 days after TCDD administration with reverse changes (slight reduction on day 4 and an over ten-fold drop on day 10) under the feed restriction regime. In H/W rats, the AHR mRNA levels were unaffected by TCDD treatment.

### 2.5. General Discussion

The extreme sensitivity difference between H/W and L-E rats, coupled with the use of feed restriction as a mimic of TCDD-induced wasting in TCDD-sensitive L-E rats, pave the way to the logic followed below in the discussion of our results. In broad terms, the most promising candidates having a causative link to TCDD-induced wasting are those variables that react discordantly to TCDD in both of these two models. However, it should still be borne in mind that any factor showing a divergent response may not only be affecting the development of the wasting syndrome but could also arise as a secondary effect of TCDD toxicity, if unrelated to body weight loss. Therefore, it is more prudent at this stage to describe the link as associative instead of causative. On the other hand, a similar response in both strains to TCDD administration implies an adaptive change or a direct effect of TCDD not causally related to its acute lethality. However, if this occurs together with a concurrent analogous change in feed-restricted L-E control rats, it will require case-wise consideration.

In the present study, there was a statistically significant decrease in body weight in both strains at 10 days. However, statistical significance is not tantamount to biological significance. The magnitude of the loss (<5%) recorded in H/W rats was vastly different from that occurring in TCDD-treated L-E rats (~30%), which translates into a difference between adaptive and fatal outcomes. Therefore, changes found only in L-E rats have potential to be involved in the mechanism of acute lethality and wasting syndrome induced by TCDD, and, conversely, changes only occurring in H/W rats may bear on resistance to TCDD toxicity.

It is worth pointing out that the focus of the present study was on the wasting syndrome, not on the mechanism(s) by which TCDD might directly alter hormonal status. Previous work has revealed that there is a wide spectrum of possible ways for TCDD to interfere with the endocrine balance, including enhanced potency of a positive regulator (ACTH [69,70]), induction of metabolism in the liver (thyroxin [71]) or extrahepatically (melatonin [72]), impaired synthesis (testosterone, corticosterone [73,74]), and induction of interactions between the AHR and hormone receptors (estrogen and androgen receptors [75]).

#### 2.5.1. Insulin and Leptin

The serum concentrations of both leptin and insulin generally followed the decrease of body weight in L-E rats and had moderate declining trends after TCDD administration in the H/W strain. However, in the case of insulin, the decrease inflicted by TCDD was more pronounced than that brought about by feed restriction. These findings are in agreement with the known reduction of blood insulin after large doses of TCDD [76,77], and with TCDD’s ability to reduce pancreatic insulin secretion at sublethal doses [76,78]. The relatively scarce data about the effects of TCDD on leptin secretion also concur with those of our current study: A small-scale experiment with TCDD-doses affecting feed intake showed an initially elevated plasma leptin concentration but then an identical downhill course in both TCDD-treated L-E rats and their pair-fed controls [79], while unchanged plasma leptin levels were reported at 24 h after a low (1 µg/kg) dose of TCDD in rats [78]. There is a paucity of research on the effects of a gradually tightening short-term feed restriction on insulin or leptin plasma levels, but a large number of studies show both hormones to diminish in response to reduced feed intake and white adipose tissue mass, although the magnitude of the response may vastly exceed that of the trigger [19,80]. For example, in rats a pronounced drop (down to about 10% of control) of both insulin and leptin plasma concentrations followed a 48-h fast, and a similar change (to about 20%) followed a 55% 12-day feed restriction that resulted in an 8% reduction of body weight [81]. Curtailing of insulin secretion is also the first physiological response to a decline in plasma glucose concentration [36]. Taken together, it appears that leptin and insulin (see [76]) concentration changes elicited by TCDD are largely secondary to reduced energy intake and do not directly explain wasting. As insulin levels already decreased on day 1 while leptin concentrations were sustained until day 10, it is conceivable that of these two key long-term regulators of energy balance in the body, at least in L-E rats insulin has a superior role. On the other hand, TCDD appears to increase insulin sensitivity in rats and mice [8], which could be reflected in our findings, as supported by the somewhat more pronounced decrease in plasma insulin in TCDD-treated *vs.* feed-restricted L-E rats. Thus, further research on the effects of TCDD on both central and peripheral actions of insulin is warranted.

In control animals, leptin levels were much lower in L-E than H/W rats. Unfortunately, we have no additional data to shed more light on the possible reason(s) for this strain difference. Albeit the chow fed to L-E rats (see 3.1. Animals and Husbandry) contained slightly more crude fat and protein than the chow fed to H/W rats, the metabolizable energy contents were almost identical. Notably, also basal insulin levels were somewhat lower in L-E than H/W rats while basal ghrelin levels exhibited a tendency towards the reverse difference. It could thus be that L-E rats are exceptionally sensitive to the negative regulators of feed intake and body energy balance. Alternatively, an inherent physiological difference is also possible, perhaps relating to various strain differences in glucose metabolism, taste responses and circadian feeding patterns [82].

#### 2.5.2. Ghrelin and Corticosterone

Ghrelin serum concentrations exhibited a rapid (starting already on day 1), progressive increase in TCDD-treated L-E rats with a delayed, curtailed response in H/W rats, and a more lenient elevation in feed-restricted controls. This change points to an additive effect of TCDD and feed intake reduction on circulating ghrelin, and, importantly, implies indirectly to a mechanistic aspect in TCDD-induced wasting syndrome. The central orexigenic influence of ghrelin on homeostatic energy balance regulation, acting through the “classical” hypothalamic NPY/AgRP—melanocortin system, is well established (recently reviewed in [31]), and it also appears to function in the non-homeostatic food reward signaling [83]. The early and progressive increase in serum ghrelin in TCDD-treated L-E rats thus suggests a correct and rapid peripheral reaction to feed intake reduction, but in association with a central “ghrelin resistance”, in support of the notion of a derangement in the central regulatory mechanisms of body weight and food intake [8]. Pertinent to this, the mRNA abundance of growth hormone secretagogue receptor (GSH-R; ghrelin receptor) was elevated in the hypothalami of L-E rats at 24 h after a high dose (50 μg/kg) of TCDD [84], while vagotomy failed to appreciably modify the effect of TCDD on feed intake or body weight in L-E or H/W rats [85]. The latter finding is noteworthy because the majority of peripheral food intake regulators exert their action exclusively or partly via the vagus nerve [28]. The substantially increased serum corticosterone concentrations on day 10 in L-E rats alone after both TCDD treatment and feed restriction do not seem likely to modify the wasting syndrome directly, and appear to fail to rescue gluconeogenesis on day 10 after TCDD administration (see below). On the other hand, hypothalamic corticotropin-releasing factor (CRF) has been related to central body weight regulation [8], and elevated CRF, adrenocorticotropin (ACTH), and corticosterone plasma levels have been reported in rats treated with a TCDD dose that down-regulates body weight [86].

#### 2.5.3. Glugacon, Glucose and Adiponectin

TCDD affected serum glucagon and glucose concentrations in a manner which binds them together and to insulin changes, and offers a possible unifying explanation. In L-E rats, serum glucagon concentration increased slightly at 4 days and then substantially at 10 days after TCDD administration, while there was a small reduction in the feed-restricted group at the latter time-point. Concurrently, there was also an approximately 30% drop in serum glucose in TCDD treated L-E rats and a smaller one in feed-restricted animals at 10 days. Glucagon secretion is controlled both by the autonomic nervous system, reacting to plasma glucose thorough both peripheral and central receptors [63], and directly by plasma insulin [38]. At 4 days, the modest increase in TCDD-treated, but not in feed-restricted, L-E rats could be explained by the slightly reduced insulin concentration that was already present on day 1, and possibly by sympathetic activation related to stress, suggested by the upward tendency in serum corticosterone concentration. The previously mentioned primary effects also appear to be at play on day 10; however, at this time-point serum insulin concentration was drastically reduced in both TCDD-treated and feed-restricted L-E rats, while in TCDD-treated rats glucose concentration was significantly more depressed and glucagon concentration substantially increased. The approximately 10-fold increase in glucagon thus appears to be unable to correct the plasma glucose level in TCDD-treated animals at this stage, whereas in feed-restricted control rats, glucose is less affected despite the fact that glucagon levels are not elevated.

The reason for this discrepancy might lie in the rate-limiting enzyme of gluconeogenesis, phosphoenolpyruvate carboxykinase (PEPCK), whose catalytic activity in the liver is markedly induced by feed restriction but less so by TCDD treatment, due to a repressing impact of TCDD on the hepatic mRNA levels of PEPCK [87,88]. Glucagon induces hepatic glucose production immediately by enhancing glycogenolysis [37] and more slowly by inducing the transcription of gluconeogenetic genes, including *PEPCK* [89]. In lethally TCDD-dosed L-E rats, liver glycogen reserves are depleted by day 6 [87]. Glucagon-induced glycogenolysis might therefore enable TCDD-treated L-E rats to maintain their serum glucose levels close to normal at 4 days, but the counteracting effect of TCDD on *PEPCK* expression would seriously impair glucagon’s regulatory function on day 10. It is important to note, however, that hypoglycemia and PEPCK inhibition do not appear to explain the mortality of TCDD-treated L-E rats [87], but they, and the stark rise of glucagon, may contribute to the development of the wasting syndrome, especially considering the anorexigenic effect of glucagon [19,27,39]. Hypoglycemia and a low hepatic glycogen level combined with low serum insulin but high glucagon and corticosterone levels might also explain, at least in part, the inability of TCDD-exposed rats to respond by eating to an acute energetic crisis caused by 2-deoxyglucose-elicited glucoprivation, and their enhanced susceptibility to hypoglycemia and lethality induced by exogenous insulin [77,90,91].

The fact that glucagon levels were progressively increased in L-E rats which at the used dose level of TCDD show severe wasting, and concomitantly either remained unaffected or even slightly diminished in both TCDD-resistant H/W rats and feed-restricted controls, associates this hormone with the wasting syndrome. If the changes seen in L-E rats had merely arisen as a consequence of their altered metabolic state, the same changes should have been discernible in feed-restricted controls, but this is clearly not the case here.

Serum adiponectin was modestly and incoherently decreased by TCDD in both L-E and H/W rats, while it did not respond to feed restriction. This suggests that adiponectin is not causally related to the wasting syndrome but its serum level appears to be slightly reduced by TCDD. In comparison, changes in adiponectin have been related to insulin resistance and metabolic syndrome in humans putatively induced by environmental dioxin exposure [92,93]. The unresponsiveness of adiponectin to feed restriction was in line with similar findings in a previous rat study [81] applying a comparable feed restriction regime (see insulin and leptin), albeit adiponectin plasma concentration was originally shown to decrease after a long-term feed restriction in mice [94].

#### 2.5.4. IGF-1and FGF-21

The TCDD-induced profuse reduction of serum IGF-1 concentration in both L-E and H/W rats seems to downplay the role of this hormone in the wasting syndrome, although the response was more rapid in the L-E strain and concomitantly larger in TCDD-treated *vs.* feed-restricted L-E rats. This is, by and large, in line with the findings of a long-term *in vivo* study in Sprague-Dawley rats [95], where the highest loading dose of TCDD, 3.2 μg/kg (complemented with maintenance doses to reach a TCDD steady-state throughout the 128-day study), induced a significant reduction in body weight gain, a decline in circulating IGF-1 levels from day 8 on, and a downward tendency in the hepatic PEPCK activity and abundance. In that study, there were no changes in circulating insulin or glucose levels, suggesting adaptation to the retarded body weight gain, and IGF-1 and PEPCK reduction to be responses that occur at lower doses than those leading to actual wasting syndrome. Circulating IGF-1 originates mainly from the liver and is produced there under GH stimulation [57,96]. However, TCDD was found to induce mixed effects (or, at most, a slight reduction) on GH plasma levels in two studies in rats [76,97], and to stimulate GH gene expression in cultured rainbow trout pituitaries [98], making a direct repression of circulating IGF-1 through severe GH depletion unlikely. On the other hand, GH receptor mRNA was decreased by 2.3-fold in the livers of TCDD-exposed L-E rats on day 10 with no change in H/W rats (Supplementary material to [99]), which may have contributed to the outcome. In food intake repression, IGF-1 production is reduced while GH levels are generally increased [61,100]. This dual response appears to be, at least partly, mediated by FGF-21 [49,60,61], linking these two metabolic hormones together.

The progressive increase of serum FGF-21 in L-E rats and a similar, but less striking, response in the H/W strain markedly deviated from the controls and feed-restricted L-E animals, in which serum FGF-21 did not reach the assay limit. Akin to IGF-1, this finding does not support a direct link between FGF-21 increase and wasting syndrome, but the TCDD-induced increase in FGF-21 might explain the somewhat more pronounced repression of IGF-1 in comparison with feed restriction, as noted above. The lack of effect of feed restriction on FGF-21 serum levels was somewhat unexpected since in mice, a 12-h fast induced a 28-fold increase in FGF-21 gene expression in the liver [53] and a 24-h fast roughly doubled serum FGF-21 levels [52]. However, in rats a more lenient (40%) six months calorie restriction did not influence FGF-21 plasma concentrations [101], and there are conflicting reports on serum FGF-21 levels in patients with anorexia nervosa [50], suggesting that both species and type of food reduction may affect the final circulating FGF-21 level. A direct, possibly AHR-mediated, effect of TCDD on FGF-21 is conceivable, since a single low (10 μg/kg) toxic dose of TCDD has been shown to induce a 3–4-fold increase in *Fgf*-*21* mRNA expression in mice [102], although *in vitro* data points to a mutually inhibitory effect between AHR and PPARα, a positive regulator of hepatic FGF-21 [103]. Another potential contributing mechanism of FGF-21 increment related to TCDD-exposed L-E rats is through glucagon, since it has been demonstrated to increase circulating FGF-21, augmented by circulating lipids [104], but independently of endogenous insulin levels [105]. Fatty acids also appear to regulate FGF-21, since in the mouse liver, they activate PPARα leading to induction of FGF-21 transcription [52,53,106]. TCDD-treated L-E rats, but not their pair-fed controls, exhibit elevated hepatic fatty acid levels [107].

Several lines of evidence demonstrate the importance of FGF-21 in glucose production and fatty acid catabolism in the liver during starvation and in ketotic states [49,51]. However, the effects of FGF-21 on adipose tissue are complex and likely depend on the precise physiological or pathophysiological context [49]. In relation to TCDD, mice devoid of Nrf2 (an AHR-activated essential mediator of induction of many antioxidative enzymes), were found to express constitutively over 300% more hepatic *Fgf*-*21* mRNA than wild-type mice, while showing markedly aggravated hepatic steatosis after TCDD treatment, which induced *Fgf*-*21* expression to the same degree as in wild-type mice [102].

#### 2.5.5. Clinical Chemistry and AHR

In L-E rats, there was a progressive increase of FFA starting on day 1 after TCDD administration and a similar but more subdued response in feed-restricted animals. The TCDD-induced circulating FFA excess implies accelerated lipolysis in the white adipose tissue and might point to increased utilization of fat for energy in TCDD-treated rats [1,108], although previous data on the influence of TCDD on β-oxidation are conflicting [109,110]. TCDD did not alter triglyceride serum concentrations either in L-E or in H/W rats, but feed restriction induced a marked reduction both on day 4 and day 10, which might indicate futile cycling of fatty acids in TCDD-treated L-E rats. In contrast, in Sprague–Dawley rats, TCDD brought about a marked increase in triglycerides at 24 h, but a decrease at 7 days [108]. As reduced generation of triglycerides by the liver is an appropriate physiological response to a severe energy deficiency (attested to by the feed-restricted controls), the inability of L-E rats to mount this response might partially account for their greater sensitivity to TCDD lethality compared with Sprague–Dawley rats. The same study [108] also offers a potential explanation for TCDD-induced changes in circulating BHB: the subdued BHB increase after TCDD-administration in comparison with feed restriction is likely attributable to a repressing effect of TCDD on mitochondrial 3-hydroxy-3-methylglutaryl-CoA synthase 2 (*Hmgcs2*), the first enzyme in ketogenesis. As to cholesterol, its progressive increase in TCDD-exposed L-E rats starting from day 1, coupled with a parallel but delayed and mitigated elevation in H/W rats, can be explained by the drastic impact of TCDD at this dose on the key enzyme of cholesterol catabolism, CYP7A1. At both 4 and 10 days after TCDD administration, *Cyp7a1* gene expression is sustained at less than 1% of control in L-E rats, whereas in H/W rats it remains at about 10% of control [111]. The expression of this gene is already plummeting by 19 h after TCDD exposure in L-E rats, which is fully in keeping with our present findings. The repression of *Cyp7a1* expression is not affected by feed restriction [112]; thus, it represents a direct effect of TCDD. Its possible contribution to the wasting syndrome warrants further studies.

The transient doubling of ALAT and progressive increase of ASAT (reaching about four-fold values *vs.* controls on day 10) in TCDD-dosed L-E rats attest to a modest injury to hepatocytes, and differs markedly from our previous findings in C57BL/6Kuo male mice [9]. In those animals, a TCDD-dose of 500 μg/kg (roughly biologically equivalent to the one used in the current study) induced abundant random focal liver necrosis on day 6, accompanied by an over 30-fold increase in plasma ALAT but only a three-fold increase in ASAT. In general, two- to four-fold ALAT increases have been proposed to indicate hepatocellular injury in rodents, and ALAT has been considered to be more liver-specific than ASAT [113], albeit a recent study found ALAT and ASAT to be equally specific for predicting chemical-induced hepatic damage [114]. All things considered, the enhanced ASAT activity in TCDD-treated L-E rats might emanate from skeletal muscle, since a previous study revealed considerably elevated serum levels of most amino acids in them [87].

Previous studies with cultured cells have established that TCDD causes a rapid and persistent decrease in AHR protein expression, most likely via the ubiquitin–26S proteasome system [115,116]. *In vivo*, AHR protein was found to diminish initially but then show a tendency towards recovery, the speed and extent of which depended on rat strain and TCDD dose [116,117]. In mouse liver, the depression persisted for at least 6 days [9]. In the present study, AHR protein was rapidly and persistently lowered by TCDD in both rat strains, while AHR mRNA expression was fostered in L-E rats alone. Hence, it appears that the ubiquitin-mediated degradation of activated AHR is a means to curtail (excessive) AHR response, while at the same time there is an attempt in L-E rats to maintain the AHR protein level constant by regulating its mRNA expression. It should be noticed that the ubiquitin–26S proteasome system is not the only means by which the AHR activity is controlled in the body. There are also specific factors induced by activated AHR, AHR repressor (AHRR) and TCDD-inducible poly (ADP-ribose) polymerase (TiPARP), which inhibit AHR actions *in vitro* [118,119]. However, their *in vivo* roles as AHR regulators are still poorly defined and at least the ability of AHRR to repress AHR activity is likely to be tissue-, cell, and context-specific [120].

The antithetical response to feed restriction, an increase at the AHR protein level and a progressive reduction at mRNA level, imply an inverse type response of AHR to an environmental stress or a physiological perturbation not involving xenobiotics. These findings substantiate the conception of AHR as both a toxicant sensor and a physiological, constantly marginally active regulator. AHR has previously been shown to play an important role in cell cycle control and liver regeneration [121,122,123], as well as in the maintenance of gut-associated lymphatic tissue [124]. The present finding on feed-restricted L-E rats suggests that it also may also be involved—either directly or indirectly—in the maintenance of body energy balance. This conclusion is supported by the reported transient retardation of body weight gain in AHR knockout mice over the first 4 weeks of life [125], surprisingly *enhanced* body weight gain in mice maintained on a high-fat diet and treated with TCDD in comparison with untreated mice on the same diet [126], and the wasting syndrome itself. Since the magnitude of TCDD-induced wasting varies among laboratory animals [1], it is presumable that also the significance of the regulatory role of AHR in energy homeostasis may be species dependent. Further research is warranted to verify these reflections.

## 3. Experimental Section

### 3.1. Animals and Husbandry

The studies were carried out in TCDD-susceptible inbred L-E and TCDD-resistant random-bred H/W male rats. The animals were obtained from the breeding colonies of the National Public Health Institute, Kuopio, and were 18–19 (L-E) or 13–19 (H/W) weeks old at the time of the experiment. They were housed individually in suspended stainless-steel wire-mesh cages and had free access to tap water and to pelleted feed (R36, Lactamin/Lantmännen, Kimstad, Sweden; H/W or Altromin 1314, Altromin, Lage, Germany; L-E). An exception to the *ad libitum* feeding was a group of feed-restricted L-E rats, for which the amount of feed given was gradually restricted to mimic the TCDD-induced reduction of feed intake (see *3.2. Experimental Design*, for the feeding regime). The reason for the different diets between the two strains was the superior palatability of Altromin feed for the L-E rats, which are more finicky and grow at a slower pace than H/W rats. The Altromin 1314 feed contained slightly more crude fat (5.0%) and protein (22.5%) than the R36 feed (4.0% fat and 18.5% protein), while their metabolizable energy contents were almost identical; 12.5 and 12.6 kJ/g, respectively. The temperature in the animal room was maintained at 21.5 ± 1.5 °C, humidity at 55% ± 10% and the lighting cycle at 12/12 h light/dark (lights on at 7.00 am). Study plans were approved by the Animal Experiment Committee of the University of Kuopio and the Kuopio Provincial Government.

### 3.2. Experimental Design

TCDD was >99% pure as determined by gas chromatography-mass spectrometry. For dosing, it was dissolved in corn oil (Sigma-Aldrich, St. Louis, MO, USA), adjusting the dosing volume to 4 mL/kg and given as a single dose of 100 μg/kg by intragastric gavage. The control animals received an equal volume of the corn oil vehicle. In addition, the feed offered to two groups of control L-E rats was gradually restricted for 4 or 10 days based on the feed consumed by L-E rats after TCDD-administration in this experiment (day 1, 18 g; day 2, 15 g; day 3, 13 g; day 4, 10 g; day 5, 8 g; day 6, 6 g; day 7, 4 g; day 8, 4 g; day 9, 2 g; day 10, 1 g). The rats were killed by decapitation at 1, 4 or 10 days post-exposure (or after 4- or 10-day feed restriction), trunk blood was collected and liver sampled at 13.00–15.30. Serum was separated and snap-frozen in liquid nitrogen together with the liver samples. All samples were stored at −80 °C until analysis. The group size was five animals for all other groups except for TCDD-exposed and control H/W rats on day 1 where the group size was four.

### 3.3. Peptide Assays

Leptin, ghrelin and glucagon serum concentrations were measured using the corresponding Bio-Plex rat assay systems (Bio-Rad Laboratories, Hercules, CA, USA), according to manufacturer’s instructions, whereas the rest of the hormones were quantified using individual ELISA assay kits as per kit directions. Insulin, adiponectin, FGF-21, IGF-1, and corticosterone levels were measured using Ultra Sensitive Rat Insulin ELISA Kit (Crystal Chem, Downers Grove, IL, USA), Rat Adiponectin ELISA Kit (Mediagnost, Reutelingen, Germany), Mouse/Rat FGF-21 Quantikine ELISA Kit (R&D Systems, Minneapolis, MN, USA), Mouse/Rat IGF-I ELISA Kit (Mediagnost), and Corticosterone rat/mouse ELISA Kit (Demeditec Diagnostics, Kiel, Germany), respectively. For FGF-21 assay, serum was diluted to 1:1, which resulted in several of the samples not reaching the assay detection limit of approximately 15 pg/mL (the serum concentration thus being less than 30 pg/mL). For subsequent statistical analyses, the FGF-21 serum levels were then given the value of the detection limit, 30 pg/mL.

### 3.4. Clinical Chemistry and AHR Analysis

Glucose, triglyceride, BHB, and FAA serum concentrations as well as the activities of serum ALAT (EC 2.6.1.2) and ASAT (EC 2.6.1.1) were determined as described in [9] with a clinical chemistry analyzer (Konelab 30i, ThermoFisher Scientific, Vantaa, Finland). Hepatic AHR protein levels were determined by the quantitative Odyssey (Li-Cor Biosciences, Lincoln, NE, USA) near-infrared Western analysis methodology according to [9], using β-actin for normalization since its protein level was unaffected by TCDD (data not shown).

AHR mRNA abundances were measured using real-time RT-qPCR with the Rotor-Gene 3000 instrument (Corbett Research, Sydney, Australia) employing an absolute quantification method based on a standard curve [7]. In brief, total RNA was isolated from liver samples with the RNeasy Mini Kit (Qiagen Hilden, Germany) per kit instructions and reverse-transcribed as described in [7]. cDNA derived from 20 ng original RNA was added for each PCR reaction and forty PCR cycles (denaturation 95 °C, 15 s; annealing 60 °C, 20 s; extension 72 °C, 20 s; acquisition 78 °C, 15 s) were run using EvaGreen qPCR Mix Plus (Solis BioDyne, Tartu, Estonia). The AHR primer sequences were: GAG-ACC-GGC-TGA-ACA-CAG-AG (L) and AGC-TCT-TGG-CCC-TCA-GGT-AG (R), and the produced amplicon length was 126 bp. The qPCR reaction efficiency based on the standard curve was 2.03 and the reaction-specific efficiency (mean ± SD), as estimated by the Rotor-Gene software, was 1.71 ± 0.01. No unspecific products were detected in the melting analysis. Since TCDD treatment is prone to affect the mRNA abundance of many control (“housekeeping”) genes in the liver [7] and resorting to only few control genes is predisposed to inherent uncertainty [127], transcript levels were normalized to the total RNA amount used for the RT reaction, as previously suggested [128,129].

### 3.5. Statistics

The data from day 1 in L-E rats and from all time-points in H/W rats were statistically analyzed by *t*-tests. One-way analysis of variance (ANOVA) followed by the Student-Newman-Keuls test was used for days 4 and 10 in the L-E strain to compare the three groups: TCDD, food restriction, and *ad libitum* control. In the case of FGF-21 (see peptide assays), non-parametric Mann–Whitney U-test was employed for days 1 and 10 in H/W rats and Kruskal–Wallis non-parametric ANOVA followed by the Mann–Whitney U-test was used for days 4 and 10 in the L-E strain. All analyses were performed separately for each time-point. The level of statistical significance was set at *p* < 0.05. The analyses were conducted with the IBM SPSS Statistics (v. 21) software (IBM Corp., Armonk, NY, USA)

## 4. Conclusions

We compared the effects of a single large dose of TCDD on the serum levels of several energy balance-influencing hormones, clinical chemistry parameters, and hepatic AHR expression in two rat strains with a 1000-fold sensitivity difference in TCDD-induced acute toxicity. TCDD affected most of the measured parameters in sensitive L-E rats, which suffered from the typical TCDD-induced wasting syndrome, while there were far fewer alterations in the resistant H/W strain. However, analyses of feed-restricted unexposed Long-Evans rats indicated several of the perturbations to be secondary to energy deficiency. Notable increases in ghrelin and glucagon occurred in TCDD-treated Long-Evans rats alone, which links these hormones to the wasting syndrome. The newly found hepatic energy balance regulators, IGF-1 and FGF-21, proved to be targets of TCDD in both strains and appeared, as earlier proposed, to function in concert. Furthermore, FGF-21 was putatively linked to increased lipolysis brought about by TCDD, and IGF-1 exhibited responsiveness to energy deficiency. Finally, we corroborated the previous finding of a divergent response of AHR protein and mRNA to TCDD treatment, and demonstrated a reverse set of changes in the AHR protein and mRNA response to TCDD and feed restriction. The alterations found in feed-restricted rats suggest a physiological role for AHR in the regulation of energy balance.

The early and progressive increase in serum ghrelin in TCDD-treated L-E rats suggests a corrective and rapid peripheral reaction to feed intake reduction accompanied by a central “ghrelin resistance”, supporting the idea of a derangement in the central regulatory mechanisms of body weight and food intake in TCDD-induced wasting syndrome. In parallel, the approximately 10-fold increase in serum glucagon combined with a very low insulin level was unable to correct hypoglycemia in TCDD-treated L-E rats at 10 days, and might thus explain, together with diminished hepatic glycogen reserves, the susceptibility of TCDD-exposed rats to acute energetic crisis and exogenous insulin. The stark rise of glucagon may even contribute to the development of the wasting syndrome through its central anorexigenic effects. The somewhat more pronounced decrease in plasma insulin in TCDD-treated *vs.* feed-restricted L-E rats and the increased insulin sensitivity also warrant further research, albeit insulin concentration changes do not directly explain wasting.

In all, the hormonal responses we recorded in TCDD-treated L-E rats are consistent with a concerted effort of the endocrine system to instigate feed intake and thereby restore the derailed energy homeostasis. Because hypophagia still persists, these peripheral signals appear to be either falsely interpreted or superseded by counteracting factors in the CNS.
